# TRIM47 promotes malignant progression of renal cell carcinoma by degrading P53 through ubiquitination

**DOI:** 10.1186/s12935-021-01831-0

**Published:** 2021-02-23

**Authors:** Jia-xin Chen, Da Xu, Jian-wei Cao, Li Zuo, Zhi-tao Han, Yi-jun Tian, Chuan-min Chu, Wang Zhou, Xiu-wu Pan, Xin-gang Cui

**Affiliations:** 1 Department of Urology, Third Affiliated Hospital of the Second Military Medical University, 200433 China Shanghai,; 2 Department of Urology, Changzhou Second People’s Hospital, Changzhou, 213000 China; 3 Nanjing University of Traditional Chinese Medicine School of Medical and Life Sciences, Nanjing, 210023 China

**Keywords:** Renal cell carcinoma, E3 ubiquitin ligase, Proliferation, P53, TRIM47

## Abstract

**Background:**

Renal cell carcinoma (RCC) is one of the most common malignant tumors originating from the renal parenchymal urinary epithelial system. Tripartite motif 47 (TRIM47) is a member of the TRIM family proteins, which has E3 ligase activity and has been demonstrated to be involved in the occurrence and prognosis of many tumors. The main purpose of this study is to explore the role and potential mechanism of TRIM47 in promoting malignant biological behavior of RCC.

**Materials and methods:**

TRIM47 mRNA and protein levels in human renal cancer and paired normal adjacent tissues were detected by qRT-PCR and Western blot. The effects of TRIM47 knockdown and overexpression in renal cell carcinoma cells on cell proliferation, invasion and xenograft tumor growth in nude mice were analyzed. The molecular mechanism was explored by mass spectrometric exploration,Western blot and immunoprecipitation assays.

**Results:**

TRIM47 promoted RCC cell proliferation in vitro and in vivo as an oncogene. Mechanistically, TRIM47 exerted an E3 ligase activity by interacting with P53 protein to increase its ubiquitination and degradation, which further promoted the malignant biological behavior of RCC.

**Conclusions:**

Our study demonstrated that the TRIM47-P53 axis played a functional role in RCC progression and suggested a potential therapeutic target for RCC.

## Background

Renal cell carcinoma (RCC) is a common malignant tumor of the urogenital system, the incidence of which is only next to that of prostate cancer and bladder cancer, accounting for 2.2% of adult malignant tumors [1]. At present, radical surgery remains the mainstay of treatment for patients with early-stage RCC [2]. However, due to the asymptomatic or covert symptoms of kidney cancer in the early stage and the lack of awareness of cancer screening, patients often missed the early diagnosis and treatment, and at the time of diagnosis, local progression had occurred, or the condition was already in the advanced clinical stage [3].

The advent of targeted drugs as adjuvant therapy and their promising therapeutic efficacy have brought new hope for patients with high-risk and locally advanced RCC patients. Tyrosine kinase inhibitors (TKIs), such as sunitinib, axitinib, perzopanib and sorafenib, are the most commonly used molecular targeted drugs for kidney cancer. They are vascular endothelial growth factor receptors (VEGFRs) that reduce tumor cell growth by targeting the tumor angiogenesis pathway [4, 5]. However, most patients using TKIs may develop drug resistance and experience tumor progression early in treatment. In addition, patients with advanced kidney cancer are not sufficiently sensitive to sunitinib [6, 7]. It is therefore necessary to further explore the complex mechanism underlying the development and progression of RCC.

Tripartite motif-47 (TRIM47) is a member of the TRIM protein family. The most important structure in this motif is a really interesting new gene (RING) domain. Proteins with a RING domain often play the role of E3 ligase activity [8–10]. TRIM47 has a RING structure and is reportedly defined as E3 ligase [11].TRIM47 was first determined to be overexpressed in astroglioma, which is located at 17q24-25. This region can be found or amplified in many other types of tumors [12], and high levels of TRIM47 expression have been reported. It is a strong prognostic factor for prostate cancer [13]. TRIM47 overexpression could promote the occurrence of non-small cell lung cancer (NSCLC) [14], and TRIM47 could play the role of E3 ligase by interacting with SMAD4 to promote SMAD4 ubiquitination and degradation, which further increased the level of CC motif chemokine ligand 15 (CCL15) and CC motif chemokine receptor 1 (CCR1), ultimately leading to poor prognosis of colorectal cancer [15]. Therefore, we believe that TRIM47 is an important proto-oncogene involved in the development and progression of various malignant tumors. However, the expression and biological function of TRIM47 in RCC have not been reported.

The purpose of this study was to further determine the role of TRIM47 in the development and progression of RCC by immunohistochemical staining of RCC specimens, construction of TRIM47-overexpression plasmids, lentiviral shTRIM47 knockdown cell line, and Crispr/cas9 technology to construct knockout methods such as TRIM47 stably transformed strains, animal experiments, mass spectrometry detection, bimolecular fluorescent complimentary experiments and co-immunoprecipitation experiments, in an attempt to gain deeper insights into the molecular biological function and mechanism of TRIM47 in RCC.

## Materials and methods

### Patients and tissue samples

Clinical specimens of kidney cancer and paired adjacent tissues were collected from 6 patients who received surgical treatment between October 2017 and February 2018 at the Department of Urology of the Oriental Hepatobiliary Surgery Hospital affiliated to the Naval Medical University (Shanghai, China). They included 4 pairs of clear cell renal cell carcinoma (CCRCC), one pair of papillary renal cell carcinoma (PRCC) and one pair of chromophobe renal cell carcinoma (CRCC). The postoperative clinical specimens of 125 patients constituted paired RCC and paired normal adjacent tissues. Clinicopathological variables including age at age, gender, tumor size, pathological types and tumor stages were analyzed (Table 1).

**Table 1:** Relationship between TRIM47 protein expression level and clinicopathological factors in RCC patients

Clinicopathological variables	No.	TRIM47 expression		P value
	Total 125	Low	High	
Gender (%)				0.828
Male	92	64 (74.4)	28 (71.8)	
Female	33	22 (25.6)	11 (28.2)	
Age (%)				0.845
< 60	73	51 (59.3)	22 (56.4)	
≥ 60	52	35 (40.7)	17 (43.6)	
TNM (%)				< 0.01**
1	83	70 (81.4)	13 (33.3)	
2	20	14 (16.3)	6 (15.4)	
3	22	2 (2.3)	20 (51.3)	
Fuhrman (%)				< 0.01**
1	20	19 (22.1)	1 (2.6)	
2	68	53 (61.6)	15 (38.5)	
3	23	11 (12.8)	12 (30.8)	
4	14	3 (3.5)	11 (28.2)	
OS (%)				< 0.01**
+	104	79 (91.9)	25 (64.1)	
−	21	7 (8.1)	14 (35.9)	

### Cell culture

Both RCC cell lines (A498, 786-O, ACHN, 769-P, Caki-1 and OS-RC-2) and normal renal cell HK-2 (ATCC, USA) were routinely maintained in RPMI-1640 (Gibco, USA), DMEM (Gibco) and McCoy’s 5A supplemented with 10% FBS (Gibco), 100 U/mL penicillin sodium and 100 mg/mL streptomycin in a humid atmosphere with 5% CO_2_ at 37 ℃.

### Immunohistochemistry (IHC)

After dewaxing, hydration and antigen repair, the renal cell carcinoma tissue chip was added dropwise with diluted rabbit anti-TRIM47 antibody (1:500, ab155549, Abcam, USA) and incubated overnight. The diluted anti-rabbit HRP and horseradish enzyme-labeled streptavidin working solution were added dropwise to the tissue chip the next day. The tissue chip was then stained with DAB chromogen and counterstained with hematoxylin, dehydrated and observed and counted under an optical microscope. The results of histochemical staining and the evaluation criteria of the staining intensity were scored as 0 for negative, 1 for weakly positive, 2 for moderately positive, and 3 for strongly positive. The evaluation criteria for positive cell frequency were as follows: < 5%, 0 point; 5–25%, 1 point; 26–50%, 2 points; 51–75%, 3 points; and > 75%, 4 points.

### TIRM47 overexpression plasmids and RNA interference

The coding sequence of TRIM47 was synthesized by TSINGKE Biological Technology (Shanghai, China), and then inserted into pcDNA3.1(+) using the Quick-Fusion cloning kit (Biotool, USA).The shRNA sequence of TRIM47 was CAAGAAGTCCTGCATATCCGT. After 293T cells were infected with the lentivirus packaging plasmid, and 769-P and A498 cells were infected with the virus for 48 h, they were observed under a fluorescence microscope.

### Quantitative real‐time PCR (qRT-PCR)

TRIZOL (Invitrogen, USA) was used to extract the total RNA, and then reverse transcribed into cDNA. Finally, SYBR Premix Ex TaqTM II (Takara, Japan) was used to quantify gene transcripts on the 7900HT fast RT-PCR system (Life Technologies Corporation, USA) and standardized with GAPDH.Primer sequence: GAPDH-F: GTCTCCTCTGACTTCAACAGCG; GAPDH-R: ACCACCCTGTTGCTGTAGCCAA; TRIM47-qF: ACGGCAGTGGACCCTTCAG; TRIM47-qR: CCAGGCAGGCGAGACAGAA.

### Western blot analysis

Total protein was first extracted using SDS-PAGE and then transferred to a PVDF membrane (Thermo, USA). Afterwards, the PVDF membrane was incubated with 1:1000 dilution of antibody: TRIM47 (ab155549, Abcam, USA), BAP1 (ab199396, Abcam, USA), P21 (ab188224, Abcam, USA), P53 (ab131442, Abcam, USA), SRC (ab47405, Abcam, USA), MET (ab51067, Abcam, USA), and c-Myc (ab39688,Abcam, USA). The membrane was washed and incubated with a 1:2000 dilution of horseradish peroxidase-conjugated goat anti-rabbit (Santa Cruz, USA). SuperSignalMT West Puico PLUS (Termo Scientific, USA) was used to develop the blot, using b-actin (ab8226, Abcam, USA) as the loading control. All experiments were performed in triplicate.

### CCK8 assays

After digestion, counting and centrifugation, overexpression cells or knockdown cells were seeded into 96-well plates with 5000 cells per well, cultured for 24 h, and finally evaluated using the Cell Counting Kit 8 (Biotool, USA). The absorbance at 450 nm was measured using an ELx800 plate reader (BioTek Instruments Inc., USA).

### Transwell invasion assay

Matrigel was placed on ice in advance to hydrate overnight, and then diluted with serum-free medium at a 1:8 ratio, followed by addition of 50 µl matrigel to the bottom of the transwell chamber. Cells were digested, counted and then inoculated onto the transwell chamber. The total amount of cells per well was 0.4 × 10^5^/mL. The bottom of the chamber is 1640 medium containing 15% FBS, which is placed at (37 °C, 5% CO_2_) for 48 h. Cells were fixed in 10% methanol solution for 30 min, stained with 0.1% crystal violet for 20 min, and washed in tap water until the background was clear. Five fields were randomly selected under the light microscope, and the number of membrane-passing cells was counted.

### Crispr/Cas9 technology

Crispr/Cas9 system was used to construct the knockout-TRIM47-769P cell. After constructing the sgRNA-cas9 expression vector and transfecting the 769P cell line, the stable transgenic strain was constructed through the lentiviral packaging system and selected by puromycin. The sgRNA sequence of TRIM47 is CGCCTGCCTGGGCGCGCTCTGG.

### In vivo xenograft assay

The experimental procedures were approved by the Second Military Medical University. Male BALB/c-nu mice (4–5 weeks old, 18–20 g) were purchased from Shanghai Laboratory Animal Company (SLAC, Shanghai). Sixteen nude mice were equally randomized into two groups: WT-769P group and KO-TRIM47-769P group. After adjustment of the concentration to 1 × 10^7^/mL, each mouse was injected with 0.2 mL cells (about 2 million cells) into the upper right thigh and observed for 3 weeks.

### Co‐immunoprecipitation

The TRIM47 sequence was inserted into the Flag-tagged vector, and the P53 sequence was inserted into the His-tagged vector to form two recombinant plasmids. The recombinant plasmid carrying the target gene and the virus packaging plasmid were co-transfected into 293T cells and then infected 769-P cells. The corresponding stable transfected cells were screened using puromycin. The His-P53 overexpression stable transfected cells and His-P53 + Flag-TRIM47 overexpression stable transfected cells were obtained. After the extraction of the immunoprecipitated protein, the antibody was combined with protein A/G agarose, and then the bound antibody was crosslinked, followed by pre-clearing of the protein sample, antigen immunoprecipitation and antigen elution before Western Blot detection was performed.

### Data analysis

Statistical data are expressed as means ± standard deviation (SD). All data were statistically analyzed and processed using SPSS (IBM SPSS Statistics 22.0) software. The experiment was repeated three times and then analyzed statistically.

## Results

### TRIM47 gene overexpression is associated with poor survival in RCC

**Fig. 1:**
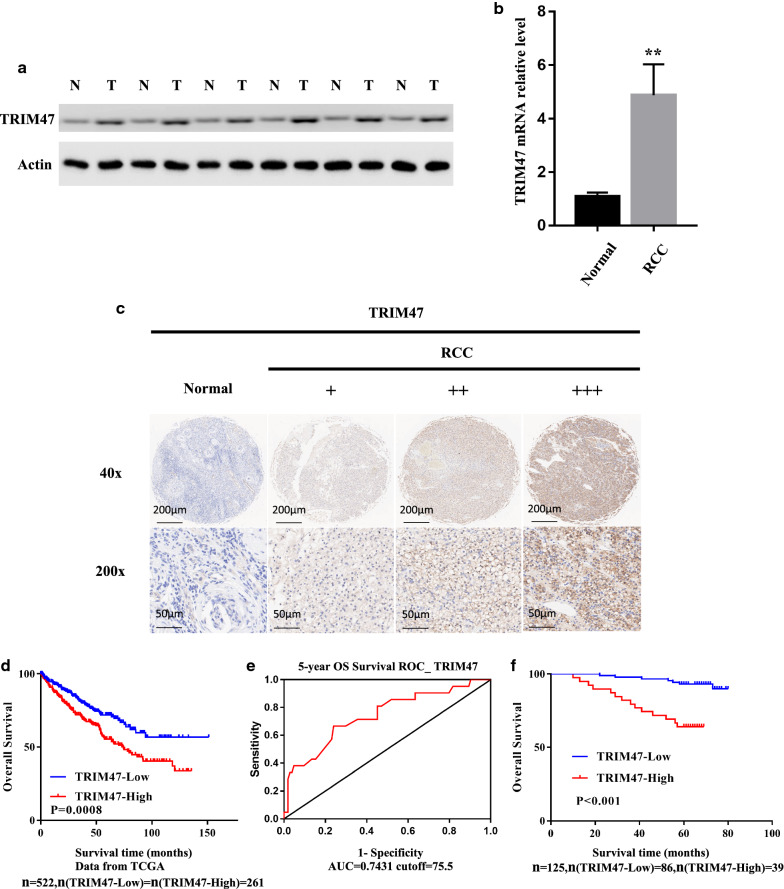
TRIM47 gene overexpression is associated with poor survival in RCC. **a**, **b **Detection of TRIM47 protein and mRNA expression levels in RCC tissues and normal adjacent tissues, P < 0.01. **c **Detection of TRIM47 expression in RCC tissues and normal adjacent tissues by immunohistochemistry. **d**The Kaplan-Meier survival analysis of RCC patients from TCGA database (P = 0.0008). **e **The receiver operating characteristic(ROC) curves for predicting the 5 years overall survival of RCC patients using TRIM47 level. **f** The Kaplan-Meier survival analysis of RCC patients from the Department of Urology, Third Affiliated Hospital of the Second Military Medical University, P < 0.001

The results of Western Blot detection of the fresh RCC and matched normal adjacent tissues indicated that the expression level of TRIM47 protein in RCC tissues was significantly higher than that in adjacent tissues (Fig. 1a). IHC staining of the tissue chips in the 125 cases of RCC and normal adjacent tissues showed that the staining degree of TRIM47 antibody in RCC tissues was significantly deeper than that in adjacent tissues, and the number of positive cells was higher (Fig. 1c) (P < 0.001). qRT-PCR detection of the fresh RCC and paired adjacent tissues demonstrated that the mRNA expression level of TRIM47 in RCC tissues was up-regulated compared with that in normal adjacent tissues (Fig. 1b) (P < 0.01). TCGA database analysis showed that RCC patients with high expression of TRIM47 in RCC had shorter overall survival (OS) and poorer prognosis than those with low expression of TRIM47 (Fig. 1d, f). Moreover, the same overall survival(OS) difference was displayed by the Kaplan-Meier survival analysis of RCC patients from the Department of Urology, Third Affiliated Hospital of the Second Military Medical University (Fig. 1e).

### Intervention of TRIM47 expression significantly affects RCC cell proliferation and invasion

**Fig. 2:**
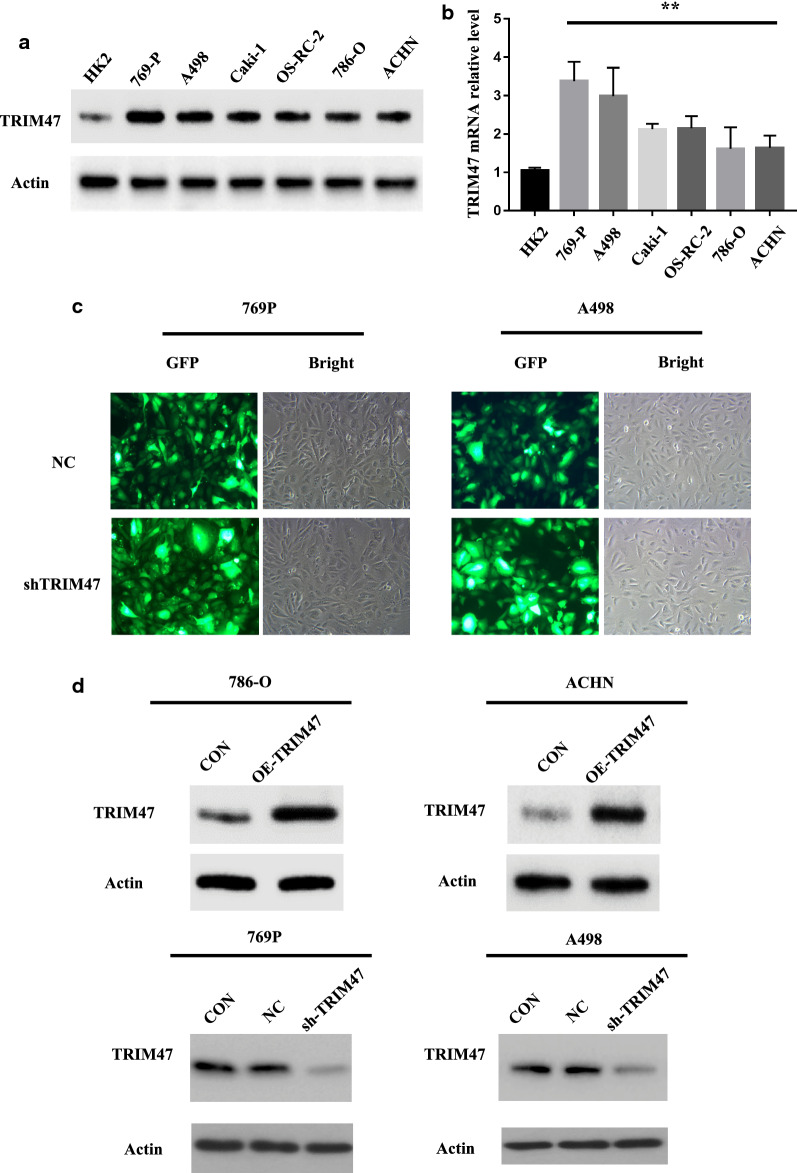
The expression level of TRIM47 in RCC cell lines and normal renal cell HK2 and the construction of RCC cell lines that interfere with TRIM47. **a** Detection of TRIM47 protein expression level in 6 different RCC cell lines and normal renal cell HK2 by Western Blot. **b** Detection of TRIM47 mRNA expression level in 6 different RCC cell lines and normal renal cell HK2 by real time PCR. **c** Lentivirus-mediated shTRIM47 was used to infect 769-P and A498 cells. **d **Western Blot was used to verify the constructed TRIM47 overexpression and TRIM47 knockdown renal cancer cell lines

**Fig. 3:**
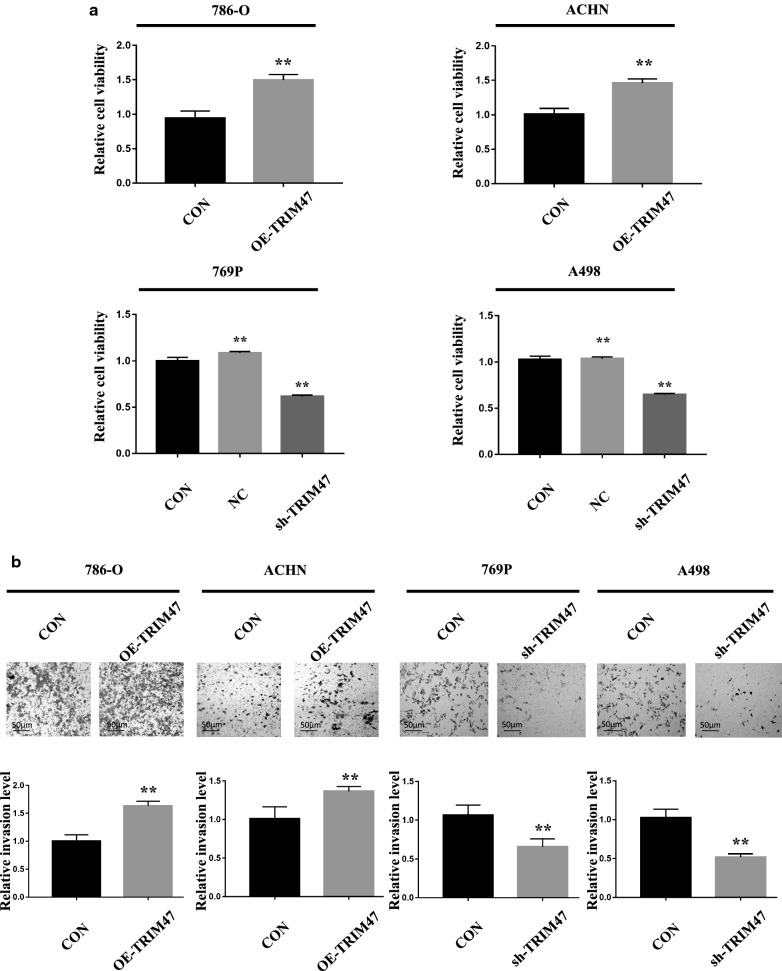
TRIM47 overexpression promoted RCC cell proliferation and invasion, and TRIM47 knockdown inhibited RCC cell proliferation and invasion. **a** Detection of the proliferation of 786-O and ACHN cells after TRIM47 overexpression and 769P and A498 cells after TRIM47 knockdown by CCK8 assay, P < 0.01. **b** Detection of the invasion of 786-O and ACHN cells after TRIM47 overexpression and 769P and A498 cells after TRIM47 knockdown by transwell assay, P < 0.01

To clarify the biological effect of TRIM47 in RCC, we firstly detected the expression of TRIM47 in 6 different RCC cell lines and normal renal cell HK2 by using Western blot and qRT-PCR, and the results showed that 769P and A498 cells highly expressed TRIM47 while 786-O and ACHN cells lowly expressed TRIM47 (Fig. 2a, b).Then the overexpression-TRIM47 cells (786-O,ACHN) and knockdown-TRIM47 cells(769P,A498) were constructed and observed by fluorescence microscope and Western blot (Fig. 2c, d). The effect of TRIM47 expression level on RCC cell proliferation and invasion was evaluated in the CCK-8 cell proliferation experiment and transwell cell invasion experiment. The result showed that the RCC cell proliferation and invasion abilities were significantly enhanced in OE-TRIM47 group, while the RCC cell proliferation and invasion abilities were significantly reduced in KD-TRIM47 group, showing a statistically significant difference (Fig. 3a, b) (P < 0.01).

### Mass spectrometric exploration of TRIM47 as the molecular mechanism underlying the malignant biological behavior of RCC cells

**Fig. 4:**
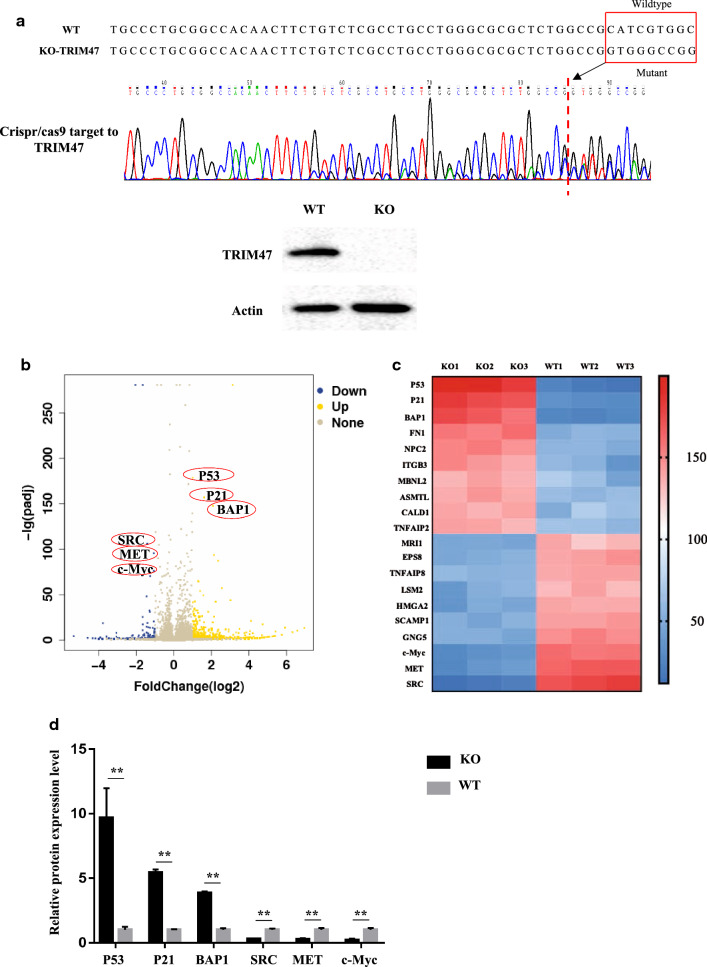
Construction of TRIM47 knockout cell line of renal cell carcinoma and analysis of mass spectrometry. **a **Sequencing and Western Blot to detect construction of TRIM47 knockout 769P cells. **b**, **c** Volcano map and heat map showing up-regulated differential proteins and down-regulated differential proteins screened out after mass spectrometry. **d **The relative protein expression level of different proteins in the two groups of cells (WT and KO), P < 0.01

**Fig. 5:**
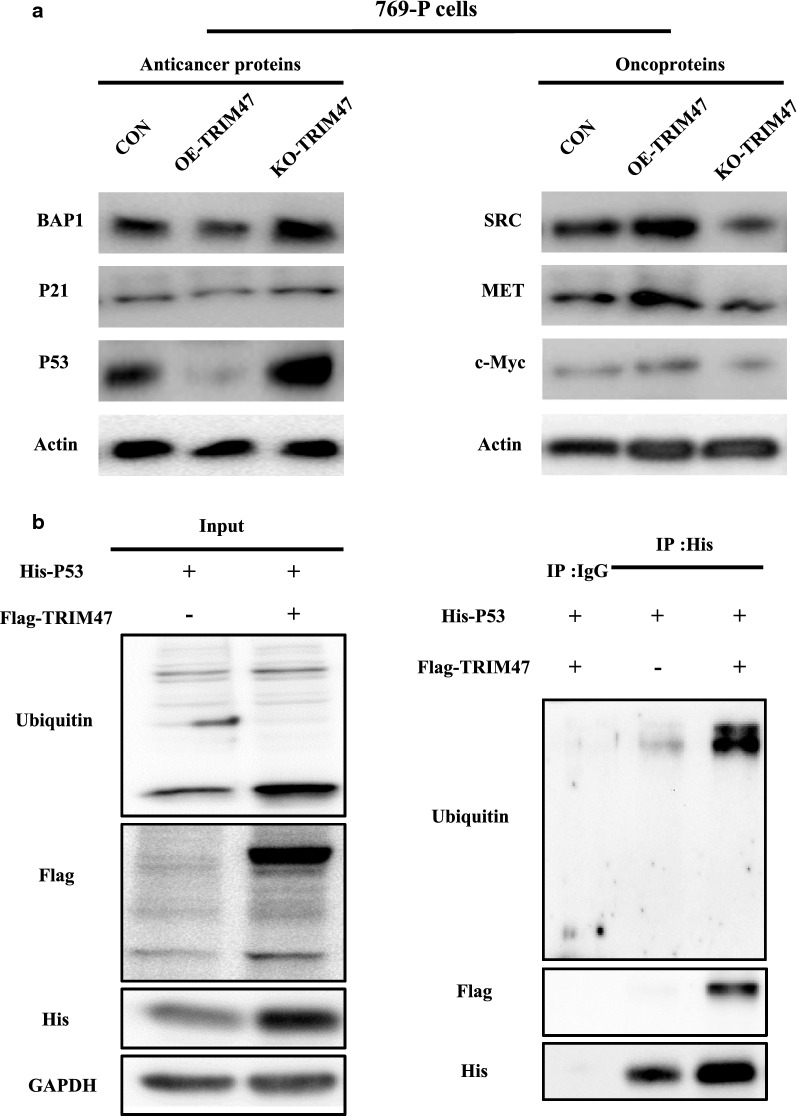
TRIM47 regulates tumor suppressor protein and oncoprotein and mediate the ubiquitination and degradation of P53 in RCC. **a **Detection of the expression of anticancer proteins and oncoproteins after TRIM47 overexpression or knockout in 769P cell line. **b** The stable His-P53 overexpression stable transfected cells and the His-P53 + Flag-TRIM47 overexpression stable transfected cells were constructed and detected by Western blot (left panel). Co-immunoprecipitation was used to detected the interaction of TRIM47 and P53 in the 769P cell line (right panel)

To understand the molecular mechanism of TRIM47 promoting the malignant biological behavior of RCC, we used Crispr/Cas9 technology to construct the knockout-TRIM47-769P cell. Then the sequencing result showed that mutations occurred near the sgRNA of TRIM47 target site, and Western blot was used to further verify the knockout efficiency (Fig. 4a). Differentially expressed proteins between KO-TRIM47-769P group and WT-769P group were analyzed by mass spectrometry (MS) (Fig. 4b–d). According to the MS results, important tumor suppressor proteins (BAP1, P21 and P53) and cancer-promoting proteins (SRC, MET and c-Myc) were detected by Western blot to observe their changes in OE-TRIM47 and KO-TRIM47 groups. The results showed that the tumor suppressor proteins were down-regulated while the tumor-promoting proteins were up-regulated in OE-TRIM47 group, and vise versa in KO-TRIM47 group (Fig. 5a).

### Detection of the interaction between TRIM47 and P53 in 769-P RCC cell line by co-immunoprecipitation assay

Given that TRIM47 plays the E3 ubiquitin ligase role, we detected the relationship between TRIM47 and P53 in RCC. Firstly, we used the 769P cell line to construct the stable His-P53 overexpression stable transfected cells and the His-P53 + Flag-TRIM47 overexpression stable transfected cells and then detected by Western blot. The ubiquitin exposed a large number of clear bands, indicating that the antibody for detecting ubiquitin protein is good, and a large number of proteins in the cell are ubiquitinated (Fig. 5b left panel). Furtherly, the protein interaction between TRIM47 and P53 in RCC 769-P cell line was detected by co-immunoprecipitation assay, and the result showed that the His antibody can pull out His-P53 itself, but the IgG does not pull out the band, indicating that the IP experiment was successful and there was no false positive. His antibody can pull out the ubiquitin of cells overexpressing His-P53, indicating that P53 is partially bound to ubiquitinated protein, and P53 is partially ubiquitinated. However, ubiquitin was obviously pulled out when His-P53 was overexpressed in Flag-TRIM47, indicating that His-P53 binds to ubiquitinated proteins more under the action of Flag-TRIM47, and ubiquitination is enhanced. These results showed that TRIM47 and P53 interacted with each other in RCC and the high expression of TRIM47 enhanced P53 ubiquitination (Fig. 5b right panel), suggesting that TRIM47 mediated the ubiquitination and degradation of P53 in RCC.

### TRIM47 knockout suppresses tumorigenicity of RCC cells in nude mice

**Fig. 6:**
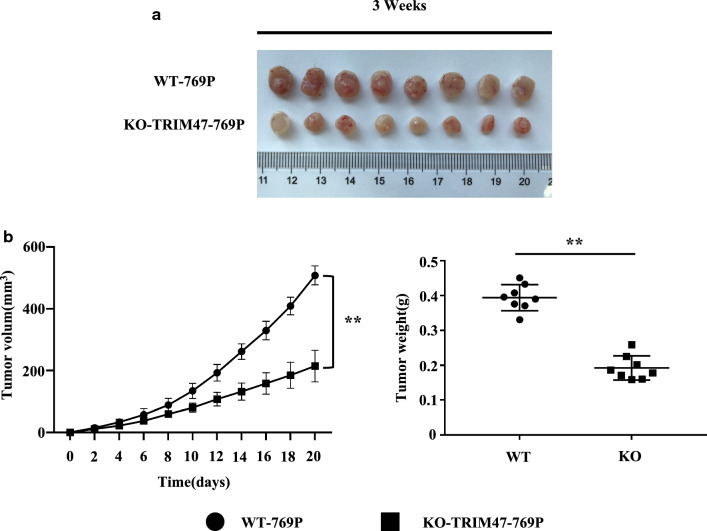
TRIM47 knockout in RCC reduces tumor growth in vivo. **a** The two groups (WT-769P and KO-TRIM47-769P) of mice were treated and the tumors were removed after 3 weeks. **b**, **c **The volume and weight of the two groups (WT-769P and KO-TRIM47-769P) of tumor tissues were measured, P < 0.01

To determine the effect of TRIM47 expression on RCC tumorigenesis in vivo, 16 nude mice were equally randomized to a KO-TRIM47-769P group and wild-type (WT)-769P group as control. After 3 weeks of the tumor-bearing experiment in mice, the tumor proliferation ability in KO-TRIM47-769P group was reduced significantly as compared with that in WT-769P group (Fig. 6a). Moreover, the tumor growth was significantly slower in mice injected with TRIM47 knockout cells and the weight of the tumor showed significantly difference between the KO-TRIM47-769P group and WT-769P group (Fig. 6b) (P < 0.01).

## Discussion

Members of the TRIM protein family are known to be involved in many biological processes, and changes in abundance or activity are related to a variety of pathological conditions, including viral infections, developmental and neurodegenerative diseases, and cancer occurrence [16]. Recent studies have shown that TRIM proteins positively and negatively regulate carcinogenesis [17, 18], and some TRIM proteins can translocate oncogenic chromosomes, where the RBCC motif is fused with another gene, and the possibility is that the RBCC motif induces translocation. It was reported that several of these proteins participated in controlling canceration by regulating the activity of P53 tumor suppressor proteins [19, 20]. Based on the currently emerging clinical evidence, ubiquitination-mediated degradation of oncogene products or tumor suppressors may be related to cancer occurrence [16]. Among the components of the ubiquitin-protein (UPS) system, E3 ubiquitin ligase, which recognizes specific substrates, is considered to be the best diagnostic and therapeutic target for cancer [21]. Most members of the TRIM protein family are endowed with E3 ligase activity due to their RING structure [22–24]. TRIM47 has a RING structure and has been shown to play a role of ubiquitination in colorectal cancer as an E3 ligase, thereby promoting tumor occurrence and metastasis [15].

IHC analysis of the RCC and matched adjacent tissue chips showed that the expression of TRIM47 was up-regulated in RCC tissues. Analysis of TCGA data showed that the expression level of TRIM47 was related to the clinical prognosis of RCC patients, and OS of the patients with high expression was lower than that in the patients with low expression. After overexpression of TRIM47, the proliferation and invasion abilities of 786-O and ACHN RCC cells were significantly enhanced. On the contrary, TRIM47 knockdown mediated by lentiviral shRNA decreased the proliferation and invasion abilities of 769-P and A498 RCC cells significantly. The results of the animal model showed that the use of Crispr/cas9 technology to knock out TRIM47 reduced the tumor-bearing ability of nude mice. The above evidence shows that TRIM47 can promote the malignant formation of RCC, suggesting that it may prove to be a potential biomarker for RCC. We then used MS to compare WT-769P with KO-TRIM47-769P. The results showed that there were many significantly differentially expressed proteins between the two types of kidney cancer cell lines, based on which, we selected 6 protein molecules with significant differences, including P53, P21, BAP1 common tumor suppressor proteins, SRC, MET and c-Myc common oncoproteins. Subsequent Western Blot assay showed showed that the protein levels of P53, P21, and BAP1 were significantly down-regulated in RCC 769-P cells after TRIM47 overexpression, and vise versa after TRIM47 knockout. At the same time, the protein levels of SRC, MET and c-Myc were increased after TRIM47 overexpression, and increased after TRIM47 knockout.

The p53 gene is the most common human tumor suppressor gene. It is located on the human chromosome 17p13.1. As its protein band is located at 53KDa, it is named P53 protein or TP53 protein. P53 protein has the ability to induce cell cycle arrest, senescence and / or apoptosis, which is the core of its anti-tumor mechanism [25, 26]. The P53 protein has complex functions. Studies have shown that it can specifically bind to DNA, and its activity is regulated by post-translational modifications including phosphorylation, acetylation, and ubiquitination [27–29]. Murine double minute 2 (Mdm2) belongs to an E3 ligase [30], which can interact with P53 itself, and regulate P53 by inhibiting its transcriptional activity, controlling its subcellular localization and regulating its protein stability [31]. Mdm2 has been shown to exert its E3 ligase function in tumors by directly binding to P53 to mediate its ubiquitination degradation, thereby promoting tumorigenesis and malignant proliferation [32–34]. Similarly, TRIM47 also has E3 ligase activity, and this study showed that TRIM47 could affect P53 protein in RCC, and the results of immunoprecipitation experiments also demonstrated that TRIM47 could interact with P53 protein in RCC, and high TRIM47 expression could enhance ubiquitination of P53 protein. These results suggest that TRIM47 could promote the generation of malignant biological behavior of RCC by mediating the ubiquitination degradation of P53. Studies showed that the expression of P21 protein was affected by the expression of P53 protein, and the expression of P53 protein in normal cells could promote its expression. In addition, P21 protein was closely related to the cell cycle. As a cyclin-dependent protein kinase inhibitor, it inhibited cyclin (the combination of Cyclin D1), and cyclin-dependent protein kinases CDK4 and CDK6 could block the cell cycle in the G0/G1 phase, and then spared time for DNA damage repair [35–38]. Therefore, the co-regulatory role of P53 and P21 in the cell cycle is the basis for affecting tumor suppression. It was found that BAP1 protein could act as a suppressor protein and played a double inhibitory role in RCC [39]. BAP1 protein is also a deubiquitinating enzyme that binds to breast cancer type 1 susceptibility protein (BRCA1) and BRCA1-related RING domain protein 1 (BARD1), which can inhibit their ability to mediate ubiquitination and autoubiquitination, thereby inhibiting tumors [40]. These findings, together with the results of our research, suggest that TRIM47 with ubiquitinase activity could promote the progression of RCC, probably by interacting with BAP1. Proto-oncoprotein c-Myc promotes the formation of RCC [41]. The increase in SRC protein in renal cancer is related to the poor survival prognosis of patients [42]. MET protein also plays an important role in the pathogenesis of renal cancer by regulating tumor growth, metastasis and angiogenesis [43]. Studies have shown that GAS6/AXL signal can regulate the invasion and metastasis of RCC through the lateral activation of MET by SRC [44]. According to our experimental results, the protein expression level of TRIM47 in renal cell carcinoma is parallel to these three renal cancer-promoting proteins (SRC, MET, c-Myc), and the higher the protein expression level of TRIM47, the stronger the invasion ability of RCC cells, which in turn promotes tumor metastasis. However, TRIM47 and its specific action mechanism have not been defined and need to be further studied.

## Conclusions

In summary, TRIM47 plays a post-translational modification role in RCC by exerting an E3 ligase activity, mediating ubiquitination and degradation of P53 tumor suppressor protein and promoting malignant progression of RCC cells at the protein level. The role of TRIM47 as a tumor-promoting molecule in promoting the malignant biological behavior of RCC is crucial. However, the specific action mechanism of TRIM47 in RCC needs further exploration. TRIM47 overexpression may prove to be a useful prognostic factor and a potential treatment target for RCC.

## Data Availability

The data sets supporting the results of this article are included within the article and its supplementary files.

## Abbreviations

RCC: Renal cell carcinoma
TRIM47: Tripartite motif 47
TKIs: Tyrosine kinase inhibitors
VEGFR: Vascular endothelial growth factor receptors
RING: Really interesting new gene
